# Cesarean Section in a Maternity Unit of a Tertiary Care Center of Nepal: A Descriptive Cross-sectional Study

**DOI:** 10.31729/jnma.5150

**Published:** 2021-04-30

**Authors:** Dhan Bahadur Shrestha, Ratna Khatri, Prakash Raj Oli, Rosy Malla, Cimona Shrestha, Roshan Khatiwada, Pratik Silwal, Prajwol Bikram Shah

**Affiliations:** 1 Department of Gynaecology and Obstetrics, Shree Birendra Hospital, Chhauni, Kathmandu, Nepal; 2 Department of Internal Medicine, Nepal Medical College Teaching Hospital, Jorpati, Kathmandu, Nepal

**Keywords:** *cesarean section*, *fetal distress*, *high-risk*, *Nepal*, *pregnancy*

## Abstract

**Introduction:**

Cesarean section is a common obstetric procedure which is done to reduce complications in high risk pregnancies. The aim of study was to find out the prevalence of cesarean section in a maternity unit of a tertiary care center.

**Methods:**

A descriptive cross-sectional study was conducted among 497 pregnant women presenting in a maternity unit of a tertiary center of Kathmandu, Nepal over a period of six months from March to August 2017 after taking ethical approval from Institutional Review Committee (Ref. 24). In this study, the prevalence of cesarean section, perinatal outcome, maternal and neonatal complications if any were observed. Data and descriptive analysis were done using Statistical Package for the Social Sciences version 22. Point estimate at 95% Confidence Interval was calculated along with frequency and percentage for binary data.

**Results:**

The prevalence of cesarean section was 171 (34.4%) at 95% Confidence interval (30.2-38.7). Most common indication for cesarean section was fetal distress 53 (31%). The maternal complications developed in 11 (6.4%) among those who delivered via cesarean delivery; Surgical Site Infection being the most common maternal complication. The neonatal intensive care unit admission rate among the newborns via cesarean section delivery was 48 (27.43%) and neonatal sepsis 14 (8%) was most common adverse neonatal outcome.

**Conclusions:**

The cesarean rate at the study center is higher than standard target rate of World Health Organization. Neonatal and maternal adverse outcome in current study were comparable with existing literatures.

## INTRODUCTION

Cesarean section (CS) is a common obstetric procedure.^1^ World Health Organization (WHO) mentions the optimal target range of CS rate to be 10-15%.^2,3^ CS rate is higher in Latin America and Caribbean countries than Asia, and Africa.^4^ In a study from 2020 showed the overall prevalence of CS in nine South and Southeast Asia was 13%.^5^ There was an increase in the population CS rate from 0.9% to 10.2% in two decades in Nepal.^6^ Previous Lower Segment Cesarean Section (LSCS), demand CS, Eclampsia are maternal and breech, cephalopelvic disproportion (CPD) are common fetal factors for CS.^7,8^ However, there are several adverse maternal and fetal outcomes with CS.^8,9^

There are few record based studies and no prospective studies from Shree Birendra Hospital. Therefore, this study was proposed and carried out.

In this study we aimed to find out the prevalence of cesarean section in a maternity unit of a tertiary care center.

## METHODS

A descriptive cross-sectional study was conducted among pregnant women presenting to the maternity unit of Birendra Hospital, Chhauni, Kathmandu, Nepal over a period of six months from March to August 2017. The study proposal was approved by the local institutional ethical review committee (IRC) of the Nepalese Army Institute of Health Sciences (NAIHS) following which study was conducted (Ref. No: 24).

The sample size was calculated using the formula,

$$\begin{array}{ll} \text{n} & ={\text{Z}}^{\text{2}}\times (\text{p}\times \text{q)}/{\text{e}}^{\text{2}}\\ & =1.96^{2}\times 0.5\times (1-0.5)/0.05^{\text{2}}\\ & =0.0096/0.0025\\ & =384 \end{array}$$

where,

n = required sample size
p = prevalence taken as 50% for maximum sample size
q = 1-p
e = margin of error, 5%
Z = 1.96 at 95% Confidence Interval

Hence, the required sample size was 384.

Adding 10% non-response rate ie. 38.4, the calculated sample size was 422.4.

A total of 497 pregnant ladies in labor were enrolled in the study using convenient sampling. With the help of semi-structured questionnaire demographic variables, obstetric history of significance, significant antenatal events, labor events, and postnatal events were recorded. Mode of delivery, perinatal outcome (gestational age at delivery, birth weight, Apgar score), and maternal and neonatal complications if any were evaluated. Informed verbal consent was taken while enrolling the individual in the study. All pregnant ladies in labor presenting to maternity unit were enrolled excluding spontaneous or induced abortions.

The collected data were entered in Statistical Package for Social Sciences version 22 and analyzed. Simple descriptive analysis performed and result were presented in appropriate tables and figures with percentage and frequencies. Point estimate at 95% CI was calculated along with the frequency and proportion for binary data.

## RESULTS

Total 497 deliveries took place during the study period. Among them, 171 (34.4%) (30.2-38.7 at 95% CI) were CS deliveries with reminder being normal and vacuum deliveries. Out of 171 CS deliveries, 111 (64.9% ) were emergency CS deliveries and rest being elective CS. Of all CS deliveries, 49 (28.7%) were among primi-gravidas women and remaining among multigravida women, with 60 (35.1%) cases having second gravida status. Out of total 171 CS deliveries, 161 (94.2%) having regualar antenatal visits in to the obstretic clinic of the study site and 88 (51.5%) cases had reached their term gestation.

**Figure 1.**
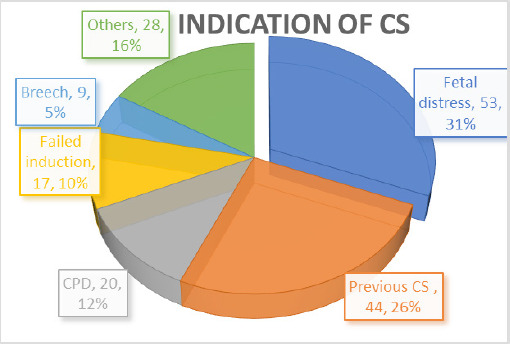
Indications of cesarean sections.

Cesarean section was performed for various indications with commonest being fetal distress 53 (31%), following previous CS 44 (26%), Cephalopelvic disproportion (CPD) 20 (12%), failed induction of labor 17 (10%), breech presentation 9 (5%) (Figure 1). Other indications were high risk pregnancies: Pregnancy Induced Hypertension (PIH), Gestational Diabetes Mellitus (GDM), subfertility treatment, Intrauterine Growth Restriction (IUGR) (Figure 1).

**Figure 2.**
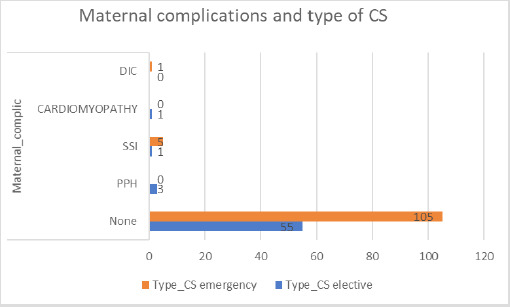
Maternal complications following cesarean sections.

Out of total 171 cesarean sections, the maternal complications were developed in 11 (6.4%) cases. The most common maternal complication was surgical site infection (SSI) 6 (3.5%) cases, more among women who had emergency CS ie. 5 (2.9%) cases. The other maternal complications were postpartum hemorrhage (PPH), Peripartum cardiomyopathy and disseminated intravascular coagulation (DIC) (Figure 2).

Of total 171 CS deliveries, 95 (54.6%) were male while 79 (45.4%) female and with 3 twin CS deliveries. 148 (84.6%) newborns were having normal weight (more than or equal to 2500 grams) rest 24 (13.7%) having low birth weight (1500-2500 grams) and 3 (1.7%) having less than 1500 grams. Neonatal complications were more in cesarean section than normal vaginal delivery, may be reflection cesarean done already among high risk cases and in which there is fetal distress also more so among emergency CS (Table 1).

**Table 1:** Type of deliveries and neonatal complications (excluding twin).

Type of delivery	Neonatal complications n (%)		Total n (%)
	No	Yes	
Normal vaginal deliveries	260 (81.76)	58 (18.23)	318 (100)
Cesarean	121 (72.03)	47 (27.97)	168 (100)
Vacuum	2 (40)	3 (60)	5 (100)
VBAC^*^	1 (100)	0	1 (100)
Total	384 (78.04)	108 (21.95)	492 (100)
Elective CS	49 (81.67)	11 (18.33)	60 (100)
Emergency CS	72 (66.67)	36 (33.33)	108 (100)
Total	121 (72.02)	47 (27.98)	168 (100)

* VABC-Vaginal birth after cesarean section

**Figure 3.**
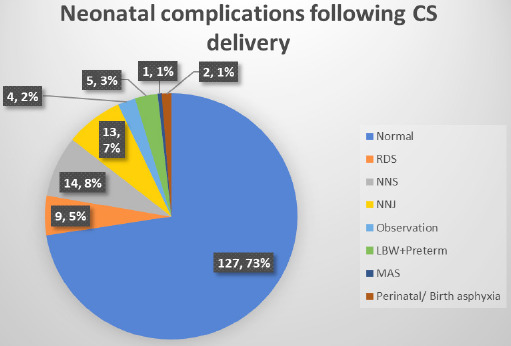
Neonatal complications following CS delivery.

Forty-eight (27.43%) newborns delivered via CS were admitted to Neonatal Intensive Care Unit (NICU) for various neonatal complications. Among different neonatal complications, the most common was neonatal sepsis (NNS) 14 (8%) followed neonatal jaundice (NNJ) 13 (7%). Other complications were neonatal respiratory distress syndrome (RDS) 9 (5%), low birth weight (LBW)/ preterm delivery 5 (3%), perinatal asphyxia 2 (1%), meconium aspiration syndrome (MAS) 1 (1%) (Figure 3).

## DISCUSSION

CS is done to reduce complications in high risk pregnancies and abnormal labor, though study showed no such improvement in perinatal mortality or morbidity.^1^ In present study the cesarean rate was 34.4% of the total deliveries with about two-third being emergency ones. The various studies from Nepal showed that the cesarean section in various government and private institutes ranges from 15 % to 81 %, higher among the private institutes.^10^ Study from Kaski, Nepal showed CS incidence of 13.3% among deliveries and influenced by age, urban residency, high education, and intrapartum symptoms.^11^ CS rate varies from one hospital to another; 4-year experience from Kirtipur hospital showed 50.9%^12^ while previous study mentioned it to vary from 10-30%.^13^ Study from China from 1991 to 2002 showed CS rate has increased from 1%-17% and CS was common among more educated women, with good income, who visited antenatal care, and giving birth equipped hospital leading to demand CS.^1,14^ Study showed CS rate has increased from 4.5 to 22.7 per 100 deliveries in 1965 to 1985 in united states (US).^15^ Sub-Saharan multi country-based study on 2011 showed CS rate of 6.2% (range 4.1-16.8%).^2^ WHO mentions optimal target range of CS rate to be 5-15%.

In this study, we found that the fetal distress (53%) is most common indication for cesarean section and it may be higher rate of its detection resulted from the use of the cardiotocography-incorporated feto-maternal monitoring. Other indications in this study are previous CS (44%), CPD (20%), failed induction (17%), breech malpresentation (9%) and others. There are numbers of maternal, fetal or feto-maternal indications for the cesarean section but the more than 85 % of them are performed for four reasons-prior cesarean delivery, dystocia, fetal jeopardy or abnormal fetal presentation.^16^ Demand CS varied from 0.3 to 14 percent is one the indication which played major role in rising trend of the cesarean section in last two decades in the Nepal also.^1,17^ Various studies from the Nepal showed that oligohydramnios (2.22-41 %), Fetal distress (09-30%), Previous CS (11-25%), CPD (6-34%), non-progress of labor (0.7-29%), fetal malpresentation (6-10%), Failed induction of labor (3-9%), PIH/pre-eclampsia/eclampsia (4-8%), Prelabor rupture of membranes (5-6%), ante-partum hemorrhage (1-2%), IUGR (2%), multifetal pregnancy (1-2%) are the common indications for the cesarean delivery.^17-20^

In present study, the maternal morbidities seen among 6.4% of total cesarean deliveries and those morbidities are: surgical site infection, postpartum hemorrhage, peri-partum cardiomyopathy and DIC. The common maternal morbidities associated with cesarean section are infection of the surgical site, hemorrhage, thromboembolism and higher maternal mortality rate compared to vaginal delivery.^16^ Commonest indication for CS being fetal distress followed by previous CS. Cesarean section is associated with chances of neonatal complications like NICU admission. The neonatal NICU admission rate in this study following Cesarean delivery is 27.43% and most common indications for the NICU admission are: neonatal sepsis, neonatal jaundice, neonatal respiratory distress syndrome, perinatal asphyxia, meconium aspiration syndrome, low birth weight. CS benefit health only in certain health conditions but also has risks like iatrogenic prematurity or respiratory distress of baby and higher maternal mortality (2-4 times) and morbidity (5-10 times) in comparison to vaginal birth.^3^

Present study has some limiations. Firstly, this study is single institutional experience based on tertiary referral center so result of present study may not be generalizable in every set up. The findings reported in this study need to be further confirmed by replicating similar studies in multiple centers with bigger patient populations. In addition, present study could not explore causality of adverse outcome due to its study design so futher prospective study is advised to confirm and explore the findings.

## CONCLUSIONS

The prevalence of cesarean section is higher than WHO target range may be because this is the only tertiary level referral center for Nepalese Army and their family. Maternal and neonatal complications observed in the center were comparable with existing literatures. In cesarean section case, the NICU admission and neonatal complications is significantly higher than normal deliveries. Careful consideration of cesarean delivery is recommended considering maternal and fetal risk and benefit of doing and not doing cesarean section in a particular case.
